# Supplementary material for: Splice-Junction-Based Mapping of Alternative Isoforms in the Human Proteome
Source: Cell Rep. Author manuscript; Available in PMC 2020 Jan 15. (PMC6961840; doi:10.1016/j.celrep.2019.11.026)
Supplement: 2 [file NIHMS1546469-supplement-2.zip › DF1/PXD006675/MitralValve/MitralValve_9_MAPKBP1_LSHQTVTR.pdf]

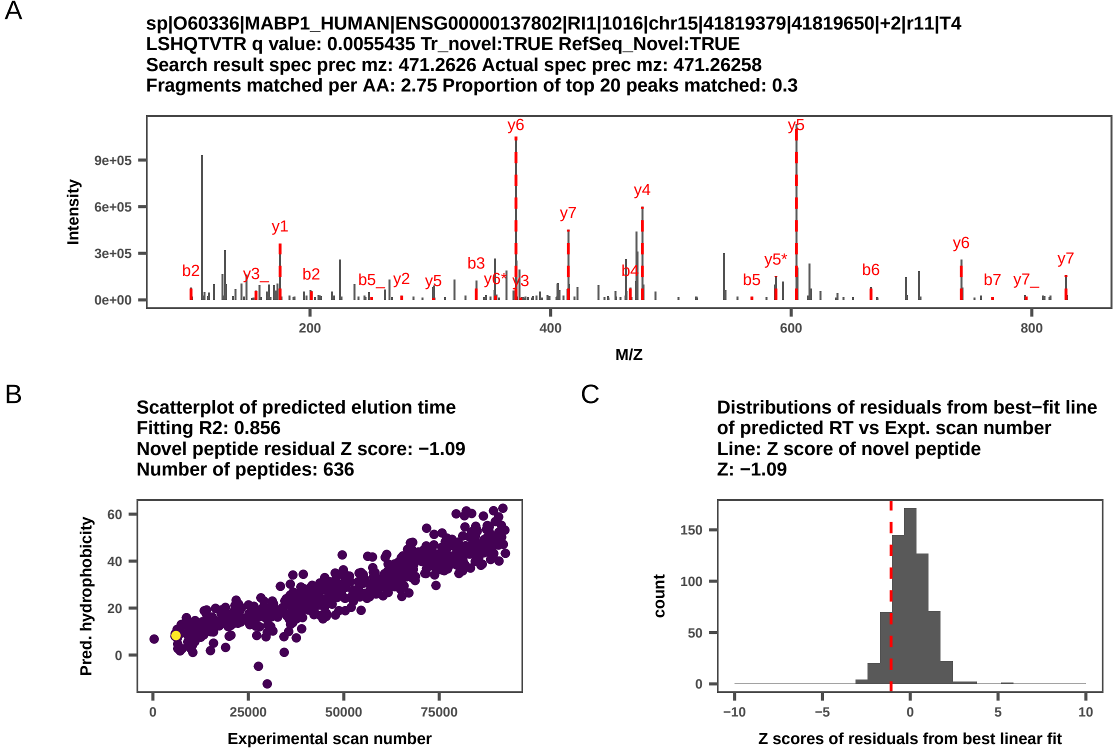
A
sp|O60336|MABP1_HUMAN|ENSG00000137802|RI1|1016|chr15|41819379|41819650|+2|r11|T4
LSHQTVTR q value: 0.0055435 Tr_novel:TRUE RefSeq_Novel:TRUE
Search result spec prec mz: 471.2626 Actual spec prec mz: 471.26258
Fragments matched per AA: 2.75 Proportion of top 20 peaks matched: 0.3
Intensity
9e+05
6e+05
3e+05
0e+00
200
400
600
800
M/Z
b2
y3_
y1
b2
b5_
y2
y5
b3
y6*
y6
y3
y7
b4
y4
b5
y5*
y5
b6
y6
b7
y7_
y7
B
Scatterplot of predicted elution time
Fitting R2: 0.856
Novel peptide residual Z score: −1.09
Number of peptides: 636
Pred. hydrophobicity
60
40
20
0
0
25000
50000
75000
Experimental scan number
C
Distributions of residuals from best−fit line
of predicted RT vs Expt. scan number
Line: Z score of novel peptide
Z: −1.09
count
150
100
50
0
−10
−5
0
5
10
Z scores of residuals from best linear fit
